# Divergent Physiological Functions of Four Atg22-like Proteins in Conidial Germination, Development, and Virulence of the Entomopathogenic Fungus *Beauveria bassiana*

**DOI:** 10.3390/jof9020262

**Published:** 2023-02-15

**Authors:** Jin-Li Ding, Hao Zhang, Ming-Guang Feng, Sheng-Hua Ying

**Affiliations:** Institute of Microbiology, College of Life Sciences, Zhejiang University, Hangzhou 310058, China

**Keywords:** *Beauveria bassiana*, Atg22, conidial germination, cytomembrane integrity, fungal differentiation, virulence

## Abstract

In yeast, Atg22 functions as a vacuolar efflux transporter to release the nutrients from the vacuole to the cytosol after the degradation of autophagic bodies. There are more than one Atg22 domain-containing proteins in filamentous fungi, but their physiological roles are largely unknown. In this study, four Atg22-like proteins (BbAtg22A through D) were functionally characterized in the filamentous entomopathogenic fungus *Beauveria bassiana*. These Atg22-like proteins exhibit different sub-cellular distributions. BbAtg22A localizes in lipid droplets. BbAtg22B and BbAtg22C are completely distributed in the vacuole, and BbAtg22D has an additional association with the cytomembrane. The ablation of Atg22-like proteins did not block autophagy. Four Atg22-like proteins systematically contribute to the fungal response to starvation and virulence in *B. bassiana*. With the exception of ∆*Bbatg22C*, the other three proteins contribute to dimorphic transmission. Additionally, BbAtg22A and BbAtg22D are required for cytomembrane integrity. Meanwhile, four Atg22-like proteins contribute to conidiation. Therefore, Atg22-like proteins link distinct sub-cellular structures for the development and virulence in *B. bassiana*. Our findings provide a novel insight into the non-autophagic roles of autophagy-related genes in filamentous fungi.

## 1. Introduction

*Beauveria bassiana* acts as an important representative entomopathogenic fungus and has been widely considered in the biocontrol of insect pests [1,2]. In eukaryotes, autophagy is a conserved process for recycling macromolecules and organelles, which are degraded in vacuole/lysosome [3]. In *B. bassiana*, this process is associated with the entire infection cycle and plays essential roles in the fungal stress response, development and virulence [4,5]. However, the roles of the autophagic process remain largely unknown in the insect pathogenic fungi.

At present, 43 autophagy-related genes (ATGs) have been identified and functionally characterized in yeast [6,7], in which 18 ATGs (e.g., ATG1 and ATG8) are considered as ‘core’ machinery genes indispensable for the autophagic process [8]. In *B. bassiana*, Atg1 (a serine/threonine protein kinase) are indispensable for autophagosome induction and expansion [4,9]. The components in the ubiquitin-like conjugation system (e.g., Atg3, Atg5, Atg7, and Atg8) are absolutely necessary for autophagy [4,10,11,12]. In addition, Atg1 directly phosphorylates the E2-like enzyme Atg3 of the ubiquitin-like conjugation system, which is indispensable for the functionality of this conjugation system [9]. Atg11 acts as an essential scaffold protein and mediates the selective degradation of mitochondria and peroxisomes [13]. In *B. bassiana* pexophagy, peroxisomes are labeled by pexoxin 14, which is recognized by Nbr1 (neighbor of BRCA1 gene 1). Then, Nbr1 recruits the targeted peroxisomes into the autophagosomes via the direct interaction with Atg8 [5]. Among the ‘core’ ATGs, the genes involved in the degradation and transportation system (DTS) differ in some degrees among fungal species. For example, the number of Atg22 significantly varies among different fungi, but at least one Atg22 is present in a fungus [6]. In yeast cells, Atg22 function as a permease responsible for the transportation of degradation products from the vacuole to the cytosol. In detail, Atg22 has functional overlaps with two other vacuolar amino acid effluxers, Avt3 and Avt4. After autophagic degradation, Atg22 mediates the efflux of leucine and other amino acids resulting from the vacuoles. The recycled amino acids maintain protein synthesis and cell viability under nitrogen starvation [14]. The homolog survey indicates that there are four Atg22-like proteins in *B. bassiana*, which is not prevalent among fungal species [6]. This implicates that *B. bassiana* might use a set of Atg22-like permeases at different stages of its lifecycle, and the action mode of Atg22-like proteins needs to be revealed at the molecular levels.

In this study, four Atg22-like proteins were functionally characterized in *B. bassiana* via the construction gene disruption and complementation mutant strain. Four proteins displayed divergent sub-cellular localizations and biological functions, including fungal differentiation, stress resistance and virulence.

## 2. Materials and Methods

### 2.1. Strains, Media and Growth Conditions

Wild type (WT) strain of *B. bassiana* ARSEF2860 (Bba2860) was obtained from the U.S. Department of Agriculture Entomopathogenic Fungus Collection (Ithaca, NY, USA),as maintained previously [13], and the fungal strains were maintained on SDAY (4% glucose, 1% peptone, and 1.5% agar plus 1% yeast extract) at 25 °C. *Escherichia coli* DH5α (Invitrogen) was cultured in a Luria-Bertani medium with the necessary antibiotics for plasmid construction. In fungal transformation, *Agrobacterium tumefaciens* AGL-1 acts as a donor strain and is cultured in YEB broth (w/v: 0.5% sucrose, 1% peptone, 0.1% yeast extract, and 0.05% MgSO_4_). Czapek-Dox agar (CzA) (3% glucose, 0.3% NaNO_3_, 0.1% K_2_HPO_4_, 0.05% KCl, 0.05% MgSO_4_, and 0.001% FeSO_4_ plus 1.5% agar) was used as the chemical defined medium in the following experiments.

### 2.2. Bioinformatic Analyses and Sub-Cellular Localizations of Atg22-like Proteins in B. bassiana

Four Atg22-like proteins have been recognized in the *B. bassiana* genome [6]. Their domain architectures were analyzed through the online portal, SMART (http://smart.embl-heidelberg.de, accessed on 15 December 2022) [15]. Phylogenetic and molecular evolutionary analyses for the Atg22-like proteins were conducted using MEGA version 5. Relationships among the Atg22-like proteins were constructed using the Neighbour joining method and the bootstrap values generated from 1000 replicates [16]. 

Cellular localizations of the Atg22-like proteins were determined as described previously [17]. All of the primers are shown in Table S1. The coding sequence was amplified with primers PLx1 and PLx2 (X: BbAtg22A–BbAtg22D) using cDNA as a template. All of the PCR experiments were performed according to the standard procedures. The resulting amplified band was cloned into pBMGS and fused with the 5′-end of the green fluorescent protein gene (GFP). The expression vector was transformed into the WT strain, and the candidate transformant was screened on the CZA with phosphinothricin (200 µg/mL). Fungal strains were grown in SDB medium (SDAY plate without agar) at 25 °C for 2 d, and the resulting mycelia were stained with 7-amino-4-chloromethylcoumarin (CMAC) indicating vacuoles or nile red indicating lipid droplets. The fluorescent signals in the mycelia were observed with a laser scanning confocal microscope (LSM 710, Carl Zeiss Microscopy GmbH, Jena, Germany). 

### 2.3. Targeted Gene Disruption

All of the *B. bassiana* disruption mutants were generated using homologous replacement coupled with a fluorescence reporter [18]. All primers are included in Table S1. The up- and down-stream flanking regions of the indicated gene were amplified using the primer pairs P_X_1/P_X_2 and P_X_3/P_X_4, respectively. The resulting fragments were sequentially cloned into the *Xma*I/*Bam*HI and *Xba*I/*Hpa*I sites of p0380-bar using the ClonExpress II One Step Cloning Kit (Vazyme Biotech, Nanjing, China), generating the gene disruption vector (p0380-bar-X). The resulting vector was transformed into the WT strain with the *Agrobacterium*-based transformation method, and the transformants were screened on CZA plates with phosphinothricin (200 µg/mL). To complement the gene loss, the full-length gene was amplified with the primer pair P_X_5/P_X_6, and the obtained fragment was inserted into the plasmid pPK2-NTC-GFP containing the nourseothricin-resistance gene [19]. For screening the complementation mutants, transformants were grown on a CZA plate supplemented with nourseothricin (50 µg/mL). All transformants were screened by PCR with the primer pair P_X_7/P_X_8, and the gene disruption mutant was further verified with the fluorescence-coupled double screening method. 

### 2.4. Visualizing Autophagic Flux in Fungal Development

Fusion protein GFP-Atg8 (GA8) was used as a marker to track the autophagic process [4]. Plasmid p0380-GA8-sur was integrated into the wild type and four gene disruption mutant strains. To visualize autophagy in the aerial mycelia, conidia of the transgenic strain were inoculated on SDAY plates and cultured at 25 °C. The aerial mycelia were sampled at 3.5 days post-incubation. As for submerged mycelia, the conidia were inoculated into SDB (SDAY without agar) and cultured for 2 d at 25 °C. The mycelial samples were stained with CMAC and examined with a fluorescent microscope.

### 2.5. Assays for Conidial Germination, Fungal Growth, and Development

To assess conidial germination, WA plates (1.5% agarose) were used as the nutrient-limited medium, while SPA plates (2% sucrose, 0.5% peptone, and 1.5% agar) were used as the nutrient-rich medium. The conidial suspension (500 µL, 5 × 10^7^ conidia/mL) was inoculated on the plates, and the germination percentage was examined at 24 h post-incubation, at 25 °C.

The fungal radial growth on the plate was assayed by replacing sucrose or NaNO_3_ in CZA with various carbon and nitrogen sources. The carbon sources (final concentration) included trehalose (3%), sucrose (3%), glucose (3%), fructose (3%), olive oil (0.5%) and oleic acid (0.2%). Nitrogen sources (final concentration) included NH_4_NO_3_ (0.5%) and gelatin (0.5%). The conidial suspension (1µL, 10^6^ conidia/mL) was inoculated on a plate. The radial growth was determined by measuring the colony diameter after 7 d of incubation at 25 °C.

The conidial production was determined on a SDAY plate. Aliquots (100 μL of 10^7^ conidia/mL) were evenly spread on agar plates and cultured for 7 d at 25 °C. Mycelial discs, 5 mm in diameter, were suspended in 0.02% Tween-80 solution. The conidial concentration was examined and used to calculate the conidial yield (conidial number per square centimeter). The fungal development under a submerged condition was assayed in SDB medium (SDAY without agar). The conidia were inoculated into SDB at the final concentration of 10^5^ conidia/mL and cultivated for 3 d at 25 °C with constant shaking. The concentration of blastospores in the media was determined using microscopic counts, and the blastospore yield was shown as the number of spores per ml of culture.

### 2.6. Assays for Membrane Integrity

Nucleus staining with SYTOX Green was applied as previously described. Cells with green fluorescence means their cytomembrane is impaired [20]. The conidia suspension was inoculated onto the SDB and cultured for 2 d at 25 °C. The resultant mycelium were collected and stained with SYTOX Green for 10 min away from light. The green fluorescence was detected under a laser scanning confocal microscope (LSM 710, Carl Zeiss Microscopy GmbH, Jena, Germany).

### 2.7. Pathogenicity Assays

For pathogenicity tests, the last instar larvae of *Galleria mellonella* were used as the bioassay hosts, and each treatment included 30–35 larvae. The fungal strains were cultured on SDAY plates for 7 d at 25 °C, and the resultant conidia were used as infectious inocula. In the cuticle inoculation assay, insects were immersed in a conidial suspension (10^7^ conidia/mL) for 10 s. In the intrahemocoel injection assay, conidial suspension (5 µL, 10^5^ cells/mL) was injected into the host hemoceol. Tween-80 solution (0.02%) was used to suspend the conidia and also as the control in the assays. The daily-recorded mortality was used to calculate the median lethal time (LT_50_) by the Kaplan-Meier method with a log-rank test for determining the difference between the paired survival trends. 

### 2.8. qRT-PCR Assays

The transcriptional analyses for the genes were performed as reported previously [21]. The wild type strain was cultured on a SDAY plate, and the mycelia were sampled at the indicated time point. The total RNA was extracted from the mycelial samples with RNAiso^TM^ Plus Reagent (TaKaRa, Dalian, China) according to the manufacturer’s protocol. The cDNA was reverse transcribed using the PrimeScript^®^ RT reagent Kit (TaKaRa) and used as templates to perform the qRT-PCR reaction on a Mastercycler^®^ EP Realplex (Eppendorf, Hamburg, Germany) cycler. All primers are shown in Table S1. The relative expression level of each gene was calculated as the relative expression of different time points over 2 d using the 2^−∆∆CT^ method [22]. Fungal 18S rRNA was as an internal reference.

### 2.9. Statistical Analyses

One-way analysis of variance (ANOVA) was applied in the comparison of the phenotypic measurements between the disruptant and the wild type or complementation mutant, and the statistical significance was determined by a Tukey’s honest significance test (Tukey’s HSD). The analyses were performed with the software of GraphPad Prism 8 (GraphPad Software, Boston, MA, USA). 

## 3. Results

### 3.1. Bioinformatic Analyses of Yeast Atg22 Orthologs in B. bassiana

**Figure 1:**
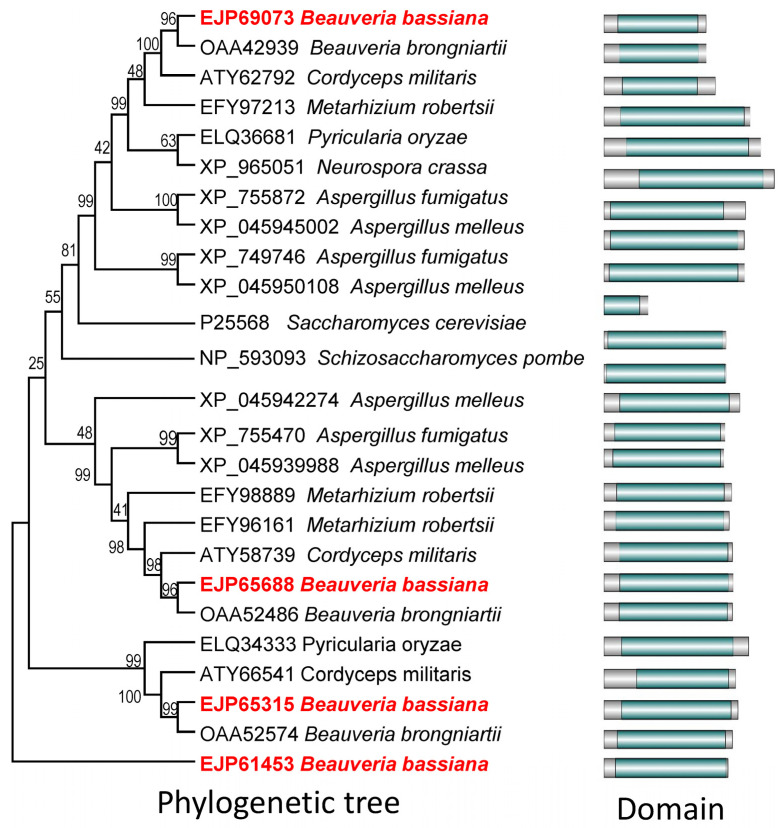
Bioinformatic analyses of Atg22-like proteins. Relationships among the Atg22-like genes were constructed by Neighbour joining method and the bootstrap values from 1000 replicates are shown at nodes. GenBank accession numbers of corresponding genes are followed by fungal species and domain architecture. Yeasts: *Saccharomyces cerevisiae*, *Schizosaccharomyces pombe*; filamentous fungi: *Aspergillus melleus*, *Aspergillus fumigatus Af293*, *Cordyceps militaris*, *Beauveria bassiana ARSEF 2860*, *Beauveria brongniartii RCEF 3172*, *Metarhizium robertsii ARSEF 23*, *Neurospora crassa OR74A*, *Pyricularia oryzae Y34*. All Atg22-like proteins have an Atg22 domain indicated with dark green. *B. bassiana* have four Atg22-like proteins, including Atg22A (EJP69073), Atg22B (EJP65688), Atg22C (EJP65315), and Atg22D (EJP61453).

Four Atg22-like proteins have been bioinformatically characterized in *B. bassiana* and are named as BbAtg22A (EJP69073), BbAtg22B (EJP65688), BbAtg22C (EJP65315), and BbAtg22D (EJP61453) [6]. All of the Atg22-like proteins in the *B. bassiana* contained a typical ATG22 domain. Phylogenetic analyses indicated four *B. bassiana* Atg22-like proteins clustered in different clades, in which BbAtg22A was clustered with yeast ATG22 (Figure 1).

To further examine the biological roles of the four Atg22-like proteins, their disruption and complementation strains were constructed by homologous recombination and ectopic insertion strategies, respectively (Figure S1). 

### 3.2. Gene Expression and Cell Biology of Atg22-like Proteins

**Figure 2:**
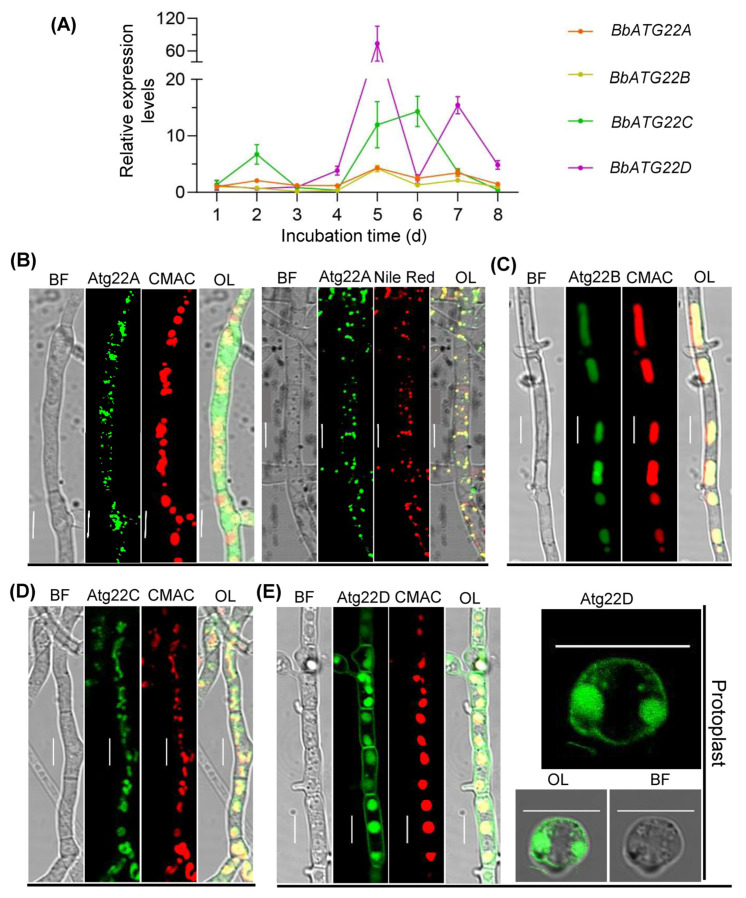
Expression profiles of four Atg22-like protein genes and sub-cellular localization of their proteins. (**A**) Relative transcriptional levels of four Atg22-like protein genes. Conidia of the wild type strain were inoculated on SDAP plates and cultured at 25 °C for 8 d. At the indicated time point, fungal cells were sampled, and the gene expression levels were analyzed with quantitative PCR. (**B**–**E**) Sub-cellular localizations of four Atg22-like proteins. Atg22-like protein gene was fused to green fluorescent protein gene, and the fusion gene was transformed into the wild type strain. The 2-day-old mycelia for the transgenic strain were harvested from SDB, and fluorescent images were examined under a fluorescence microscope. Vacuole-specific dye (CMAC) and lipid-specific dye (Nile Red) are showed in red. Scale bars: 5μm. In panel E, the association of Atg22D with cyto membrane was verified in a protoplasting experiment.

The transcriptional analyses indicated that the four BbAtg22 protein genes displayed dynamic expression profiles. The wild type strain was cultured on SDAY plates at 25 °C. The relative expression level of *BbATG22D* reached the maximal value at five days post incubation, and that of *BbATG22C* reached the maximal value at six days post-incubation (Figure 2A).

The Atg22-like protein genes were fused to a GFP gene individually and transformed into the wild type strain. The fungal strains were cultured in SDB for two days. As illustrated in Figure 2B, BbAtg22A does not overlap with the vacuoles, which were labeled by CMAC (a vacuole specific dye), but completely overlaps with the lipid droplets labeled by Nile Red. The green fluorescence signals of BbAtg22B and BbAtg22C were completely consistent with those emitted by the CMAC (Figure 2C,D). BbAtg22D showed strong GFP signals along the cell peripheries and in the vacuoles. After removing the cell walls in the protoplasting experiment, the green fluorescence still remained in the cell periphery and vacuoles (Figure 2E). These results suggest that these four Atg22-like proteins displayed the distinct sub-cellular locations.

**Figure 3:**
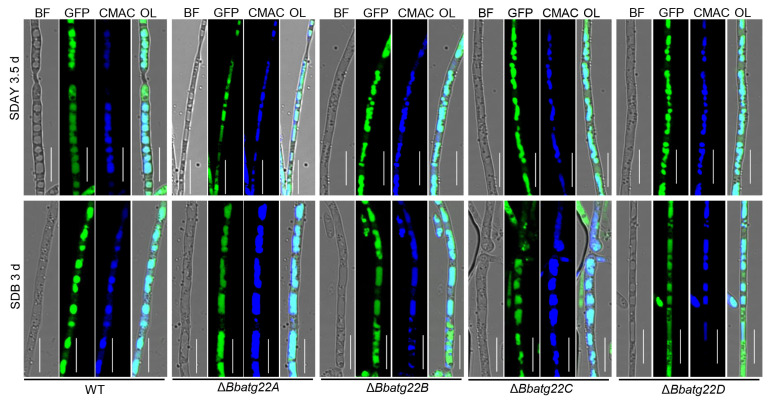
Autophagic flux during fungal development. A fusion gene of GFP-BbATG8 was integrated in the wild type and four disruptants to track the autophagic process in mycelia during development. Transformant were cultured on SDAY and in SDB for 3.5 d and 3 d, respectively. Vacuoles were indicated by staining with fluorochrome. Green signals overlapped with blue signals from CMAC. Scale bars: 5 μm.

As illustrated in Figure 3, autophagic flux was indicated with the fusion protein GFP-Atg8. Green signals were co-localized with blue signals from the vacuole-specific dye in the wild type strain, as well as the four disruptants. This result indicates that the ablation of the four Atg22-like protein genes did not block the autophagic flux.

### 3.3. Atg22-like Proteins Had Different Contributions to Cell Membrane Integrity 

**Figure 4:**
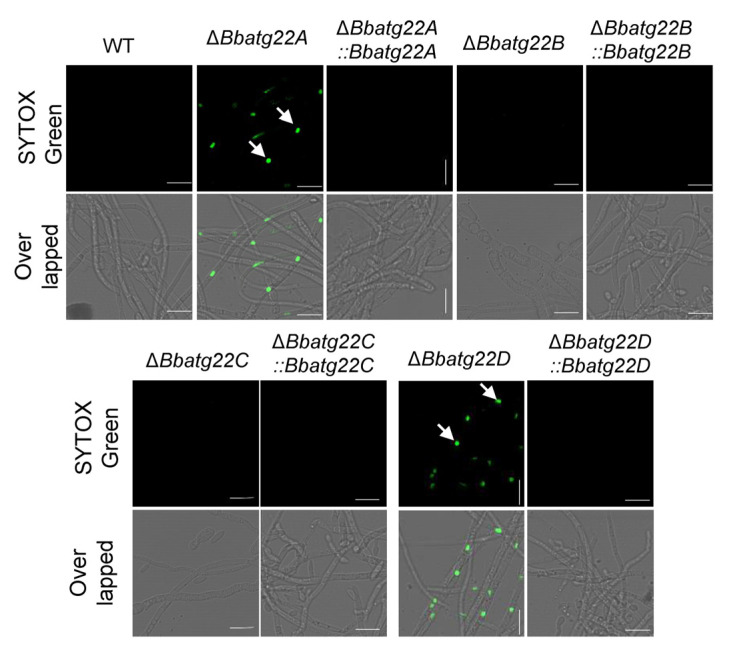
Assays for membrane integrity. Conidial suspension was inoculated into SDB and cultured at 25 °C for 2 d. The resultant mycelia were stained with SYTOX Green (nucleus-specific dye), and the fluorescent signals were examined under a fluorescent microscope. Green signals were observed in most nuclei in mycelia of Δ*Bbatg22A* and Δ*Bbatg22D* (indicated with arrows). Scale bars: 10 μm.

Cytomembrane integrity was determined by a SYTOX staining assay (Figure 4). Green fluorescent signals were observed in most cells of the Δ*Bbatg22A* and Δ*Bbatg22*D mutant strains; however, only less than 10% of the wild-type conidia were stained by this dye. The disruption of *BbATG22B* and *BbATG22C* did not significantly increase the percentage of stained cells. This indicated that the loss of BbAtg22A and BbAtg22D impaired the cytomembrane integrity.

### 3.4. Atg22-like Proteins Is Required for Conidial Germination under Nutrient-Limitation Condition

**Figure 5:**
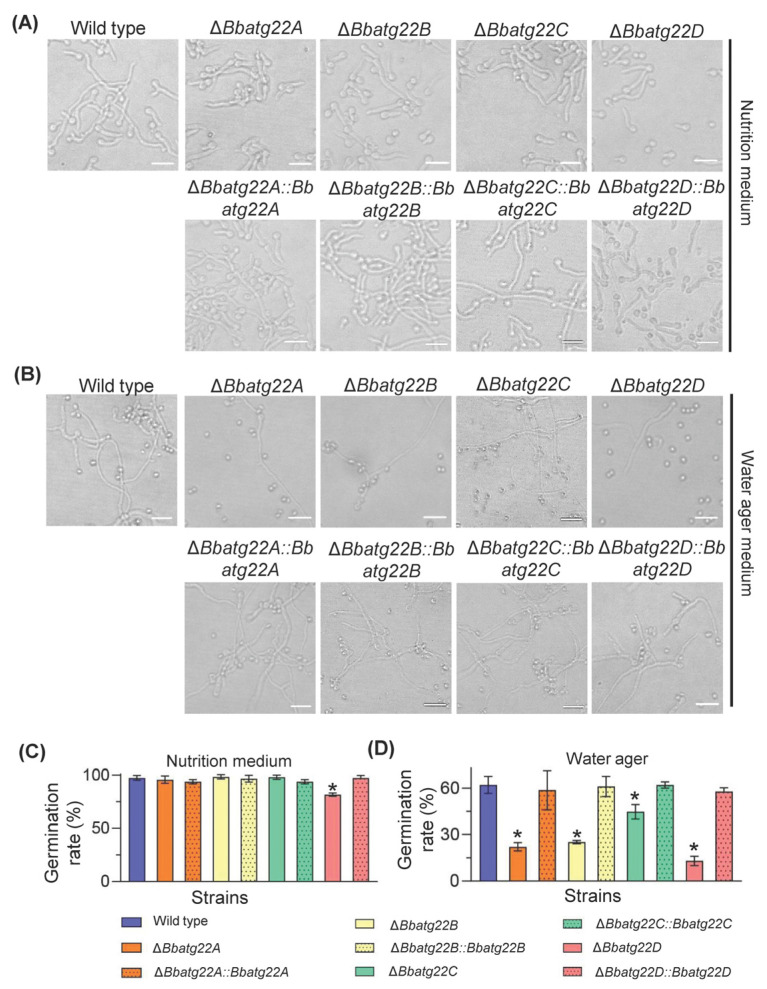
Impacts of gene loss on conidial germination of *B. bassiana*. Aliquots of 500 µL suspension (5 × 10^7^ conidia/mL) were inoculated on the nutrient medium SPA and water agarose plates and incubated at 25 °C. Incubation time for these two kinds of media was 12 and 24 h, respectively. Microscopic images of germlings formed on nutrient medium and water agarose plates are showed in panel (**A**) and (**B**), respectively. Germination percentages on these two kinds of media are showed in panel (**C**) and (**D**), respectively. Scale bars: 10 μm. Asterisks on the columns indicate a significant difference between the disruption mutant and the wild type or complemented strains [Tukey’s honestly significant difference (HSD), *p* < 0.05]. Error bars: standard deviation.

The conidial germination was assayed on GM and WA plates, which represented nutrient-rich and -limitation conditions, respectively (Figure 5A). On the GM plates (Figure 5B), after an incubation of 12 h, Δ*Bbatg22A*, Δ*Bbatg22B* and Δ*Bbatg22C* did not exhibit a significant difference in the germination rate when compared with the wild type strain. However, the germination percentage for Δ*Bbatg22D* was 81.33 ± 1.25% (mean ± standard deviation (SD)), which was 16.00% lower than that of the wild type (97.33 ± 1.89%) (Figure S2). On the WA plates (Figure 5C), at 24 h post-incubation, the germination rates for Δ*Bbatg22A* (22.00 ± 2.16%), Δ*Bbatg22B* (25.00 ± 0.82%), Δ*Bbatg22C* (44.67 ± 3.86%) and Δ*Bbatg22D* (13.00 ± 2.45%) were significantly lower than that of the wild type (62.00 ± 4.55%) with decreases of 64.52%, 59.68%, 27.96%, and 79.03%, respectively. There was no significant difference between the complementation strains and the wild type.

### 3.5. Atg22-like Proteins Are Involved in Fungal Development

**Figure 6:**
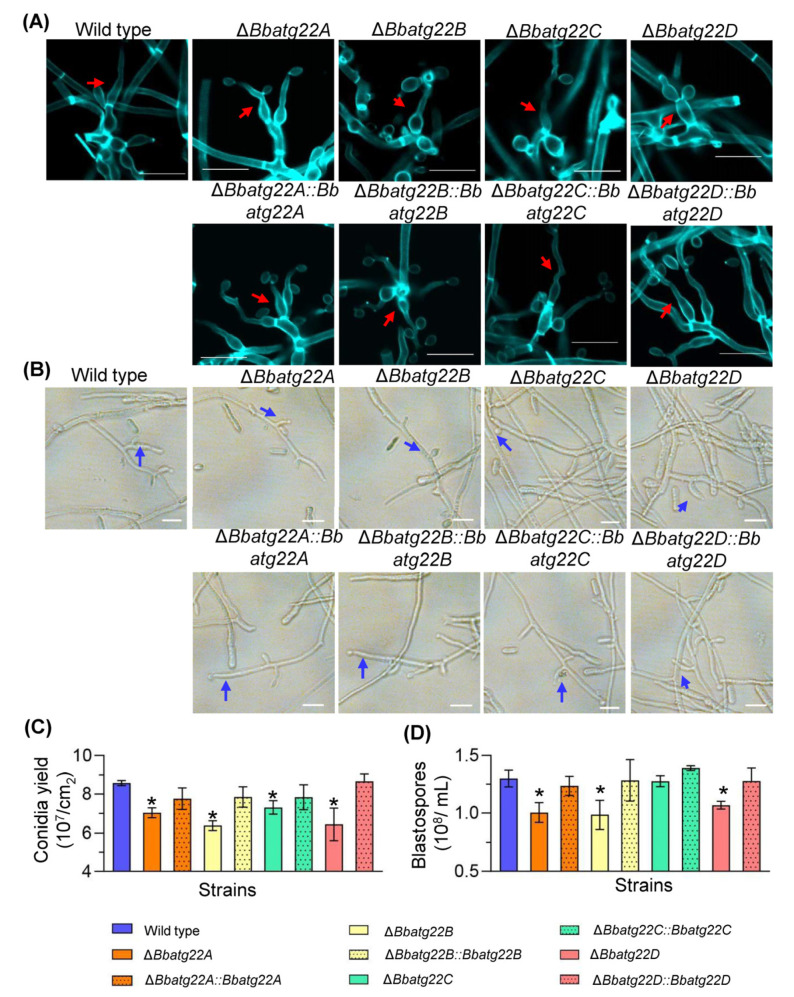
Assays for asexual development in *B. bassiana*. For aerial development, conidia of the indicated strain were inoculated on SDAY plates and cultured at 25 °C. (**A**) Microscopic view of conidiophores was recorded at 3.5 d post-incubation (dpi). Under submerged condition, conidial suspension were inoculated into SDB and cultured at 25 °C for 3 days, and then blastospore-forming structures were recorded as microscopic images (**B**). Conidial yield was examined at 7 dpi (**C**), and blastospore production was quantified at 3 dpi (**D**). Red and blue arrows indicate the conidium- and blastospore-forming structures, respectively. Scale bars: 10μm.Asterisks on the columns indicate a significant difference between the disruption mutant and the wild type or complemented strains [Tukey’s honestly significant difference (HSD), *p* < 0.05]. Error bars: standard deviation.

To determine the roles of the Atg22-like genes in nutrient utilization, the fungal growth was evaluated on different carbon or nitrogen sources. After a seven-day incubation at 25 °C, the disruption mutants showed no significant reduction in their colony diameter when compared with the wild type (Figure S3). Conidial production was examined by spreading 100 μL conidial suspension (10^7^ conidia/mL) on a SDAY plate, incubated at 25 °C. The microscopic examination indicated that the Δ*Bbatg22B*, Δ*Bbatg22C* and Δ*Bbatg22D* mutant strains produced significantly enlarged conidia-forming structures at 4 d post-incubation, and there was no significant difference between the wild type and the Δ*Bbatg22A* mutant strains (Figure 6A).After a seven day incubation at 25 °C, the conidial yields of Δ*Bbatg22A*, Δ*Bbatg22B*, Δ*Bbatg22C*, and Δ*Bbatg22D* were reduced by 18.09%, 25.67%, 14.79%, and 24.94%, respectively, when compared with that of the wild type strain (8.57 ± 0.11 × 10^7^ conidia/cm^2^) (Figure 6C). No significant differences were observed for the conidia-forming structures and conidial yield between the wild type and complemented strains.

After three days of incubation in the SDB liquid medium (Figure 6B), the blastospore-producing structures of four gene disruption mutants became shorter than those of the wild type. Except for Δ*Bbatg22C*, the blastospore yields of the other three disruptants were significantly lower than that of the wild type (1.30 ± 0.06 × 10^8^ spore/mL). The yield of Δ*Bbatg22A* decreased to 1.00 ± 0.07 × 10^8^ spore/mL; Δ*Bbatg22B* decreased to 0.99 ± 0.10 × 10^8^ spore/mL; Δ*Bbatg22D* decreased to 1.06 ± 0.03 × 10^8^ spore/mL (Figure 6D). 

### 3.6. Atg22-like Proteins Significantly Contributes to Fungal Virulence

**Figure 7:**
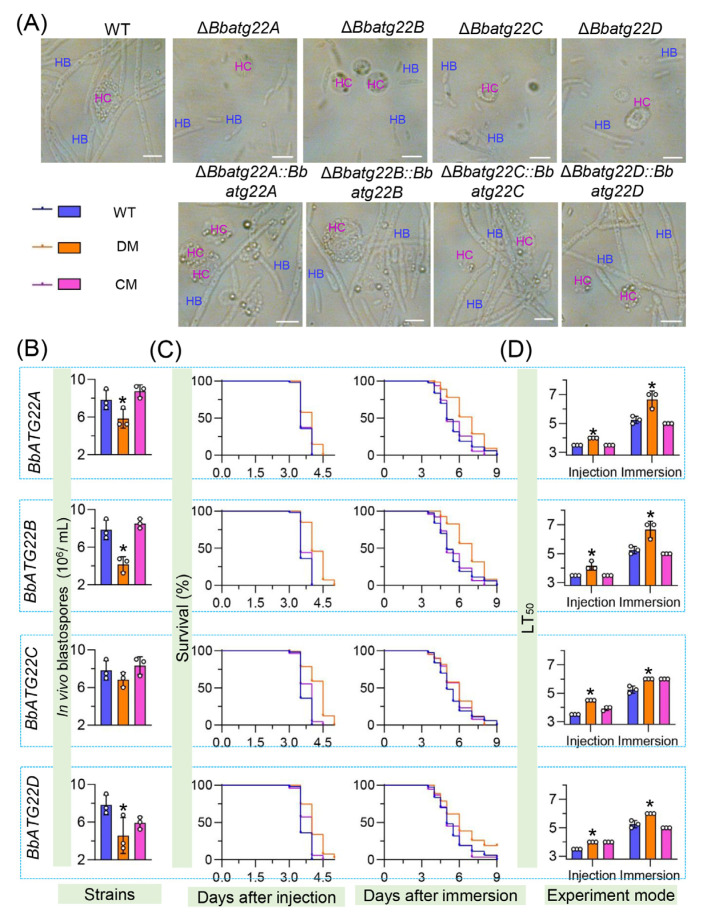
Assays for fungal pathogenic growth and virulence. To evaluate fungal pathogenic growth, conidia (500 cells) were injected into the hemoceol of *Galleria mellonella* larvae and cultured at 25 °C. Microscopic view (**A**) and production (**B**) of in vivo blastospore (hyphal body) were examined at 3.5 d post infection. Fungal virulence was determined by intrahemocoel injection of 500 conidia per larva and immersing the hosts in a 10^7^ conidia/mL suspension, respectively. The survival percentage (**C**) was recorded daily and used to calculate the median lethal time (LT_50_) (**D**) using Kaplan–Meier analyses. HB: hyphal body; HC: insect hemocytes. WT: wild type; DM: gene disruption mutant; CM: complementation mutant. *: *p* < 0.05 for Tukey’s HSD tests. Error bars: standard deviation. Scale bars: 10 μm.

Fungal proliferation in the host hemoceol was determined three days post infection (Figure 7A). The wild type and complemented strains generated plenty of yeast-like hyphal bodies, and there was no significant difference in the morphology among these strains. The yield of the hyphal body decreased by 25.53%, 46.81% and 41.48% in Δ*Bbatg22A*, Δ*Bbatg22B*, and Δ*Bbatg22D*, respectively, when compared with that of the wild type (7.88 ± 0.85 × 10^6^ cells/mL). The yield of Δ*Bbatg22C* was slightly lower than that of the wild type, but it was not statistically significant (Figure 7B). These results indicated that the loss of four Atg22-like proteins resulted in the impaired pathogenic growth in the host hemoceol. 

Conidial virulence for each strain against the greater wax moth *Galleria mellonella* was assayed by two methods of intrahemocoel injection and topical cuticle inoculation. As illustrated in Figure 7C, the survival percentage decreased with the incubation time. In two kinds of bioassay, excluding Δ*Bbatg22D*, the other fungal strains could kill all the insect hosts. The LT_50_s for the wild type were 3.50 and 5.25 days in the injection and cuticle inoculation methods, respectively. In the intrahemocoel injection, the LT_50_ values for the four disruptants (Δ*Bbatg22A*, Δ*Bbatg22B*, Δ*Bbatg22C* and Δ*Bbatg22D*) were 4.00, 4.17, 4.17, 4.50, and 4.00 days, respectively. In the cuticle inoculation bioassay, the LT_50_ values were prolonged by 1.42, 1.42, 0.75, and 0.75 days, respectively, when compared with that of the wild type (Figure 7D). Apparently, the deletion of the Atg22-like protein genes greatly reduced the virulence of *B. bassiana*, indicating the essentiality of Atg22-like proteins for the fungal virulence.

## 4. Discussion

In yeast, Atg22 functions as a transporter to release the degradation products from the vacuoles into the cytoplasm when autophagy is activated [14]. In contrast to yeast, most filamentous fungi have one to four Atg22-like proteins [6]. In this study, four Atg22-like proteins were characterized in *B. bassiana* and display distinct sub-cellular localizations, in which BbAtg22A has the highest similarity to yeast Atg22, but their sub-cellular localizations were significantly different. These findings suggest that the Atg22 domain is not a determinant factor for the sub-cellular localization of Atg22-like proteins. Similar to the homolog in yeast, the four Atg22-like proteins do not contribute to biogenesis of autophagosomes, but play different roles in conidial germination, asexual development and virulence, as discussed below.

Fungal virulence is determinant for the potential of entomopathogenic fungi as biocontrol agents [23]. In *B. bassiana*, four Atg22-like proteins are involved in the fungal virulence. The involvement of the Atg22C homolog in fungal virulence has also been revealed in another *B. bassiana* strain by insertional mutagenesis [24]. Conidial germination is indispensable for the infection initiation caused by the entomopathogenic fungi [20]. As has been revealed, four proteins systematically contribute to conidial germination under the nutrient-limited condition. *B. bassiana* conidia accumulate a plethora of nutrients, including carbohydrates and lipids/fatty acids [20,25]. The surface of the host cuticle is an oligotrophic environment. *B. bassiana* conidia mobilize the reserved nutrients for germination and the invasive growth via autophagy [4,13]. Considering the different sub-cellular localizations, four Atg22-like proteins are involved in the nutrient mobilization in different manners in *B. bassiana*. BbAtg22A, localizing in the lipid droplets, might contribute to lipid metabolism for conidial germination. In yeast, Atg22 is an integral membrane protein localized on the vacuole membrane and recycles amino acids from the vacuoles under the starvation condition [14]. BbAtg22B and BbAtg22C localize in the vacuole and might function similarly to yeast Atg22 to release nutrients from the vacuole to the cytosol. BbAtg22D has an additional association with the cytomembrane, which indicates that this protein might contribute to assimilating nutrients from ambient environments. These findings suggest that Atg22- like proteins play comprehensive roles in nutrient acquisition.

In the host hemocoel, *B. bassiana* undergo dimorphic transmission and develop into yeast-like hyphal bodies (*in vivo* blastospore) [26]. The dimorphism between hyphal and yeast-like forms is essential for fungal pathogenesis [27]. Excluding BbAtg22C, the other three Atg22-like proteins in *B. bassiana* contribute to blastospore formation in the liquid media and host hemolymph. In *B. bassiana*, other genes related to lipid metabolism are associated with morphological transition, including acetyl-coenzyme A (CoA), synthetase 2 (Acs2) [28], and sterol carrier protein 2 (Scp2) [29]. BbAtg22A and BbAtg22D are required for cytomembrane integrity, which is critical for fungal virulence [20,21]. BbAcs2 contributes to maintaining the morphology of the lipid droplet and virulence in *B. bassiana* [28]. Thus, BbAtg22A and BbAtg22D are involved in fungal virulence in the intrahemocoel injection assay, which might be a combined effect of their roles in fungal dimorphism and membrane integrity. In addition, the four Atg22-like proteins perform different influences on conidiation. *B. bassiana* develop the conidiation process to generate conidia, which facilitates fungal dispersal and initiates the follow-up infection cycle [30]. Autophagy is an efficient transport system in which the nutrients are transferred through the tubular vacuoles during conidiation [31] and has been significantly linked to conidiation in *B. bassiana* [4,13]. In *Fusarium oxysporum* (a plant pathogenic pathogen), Atg22 is required for hyphal development and conidiation [32]. These results reinforce the idea that the nutrient assimilation is essential for conidiation in the filamentous fungi.

## 5. Conclusions

Taken together, the four Atg22-like proteins display distinct sub-cellular localizations and play different roles in fungal differentiation, the response to starvation stress, and virulence in *B. bassiana*. These findings suggest that filamentous fungi evolve more Atg22-like proteins to adapt to different ecological habitats. This study improves our understanding of the roles of Atg22-like protein in filamentous fungi beyond its conserved roles in autophagy.

## Data Availability

Not applicable.

## Supplementary Materials

The following supporting information can be downloaded at: https://www.mdpi.com/article/10.3390/jof9020262/s1, Figure S1: Gene disruption and complementation in *B. bassiana*. Figure S2: Conidial germination of gene disruption mutants. Figure S3: Effects of the gene loss on vegetative growth of *B. bassiana*. Table S1: Primers used in this study.
