# Supplementary material for: Divergent Physiological Functions of Four Atg22-like Proteins in Conidial Germination, Development, and Virulence of the Entomopathogenic Fungus Beauveria bassiana
Source: J Fungi (Basel). 2023 Feb 15;9(2):262. doi: 10.3390/jof9020262 (PMC9959203; doi:10.3390/jof9020262)
Supplement: Supplementary file 1 [file jof-09-00262-s001.zip › jof-2180800-supplementary.pdf]

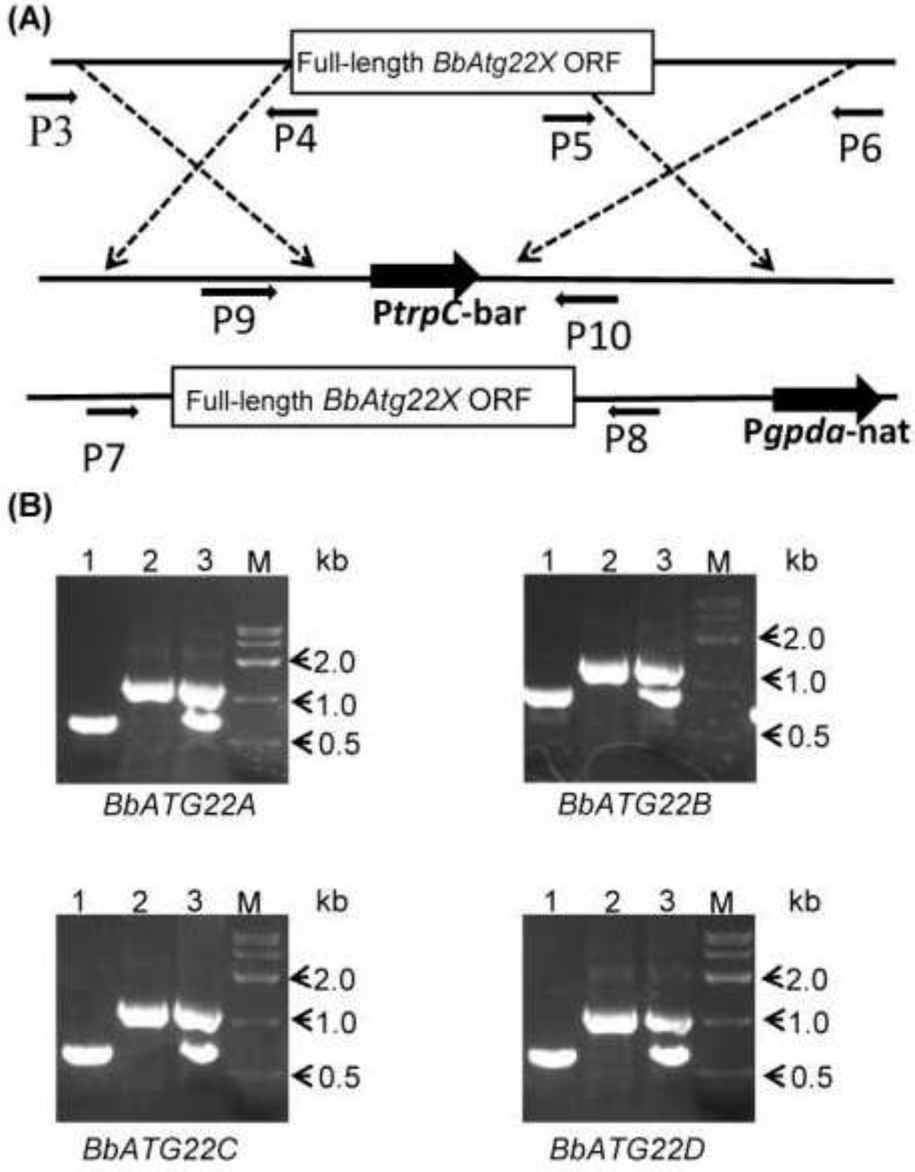

Figure S1. Gene disruption and complementation in B. bassiana. (**A**) Schematic diagram of the homologous recombination for gene disruption in fungal genome. The correct gene disruption and complemented mutants were screened by PCR reaction (**B**). In panel (**B**), lane 1, 2 and 3 are the wild-type, gene disruption and complementation mutant strains. Lane M: DNA marker. BbATG22X: BbATG22A through D. P1-P10: primer set for gene disruption and complementation of the indicated gene.

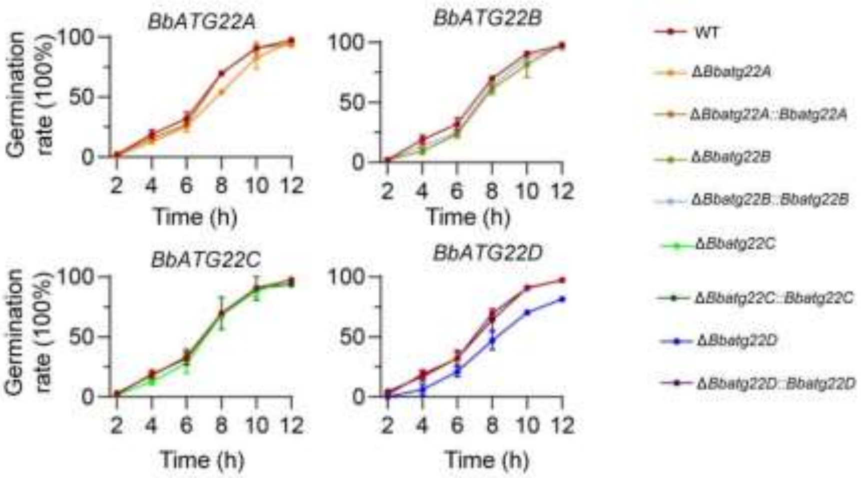

Figure S2. Conidial germination of gene disruption mutants. The wild-type (WT), gene disruption and complementation mutant strains were cultured on SDAY plates, and conidia were sampled at 7d. The sampled conidia were inoculated on nutrient-rich plates (SPA) and incubated at 25 °C. Conidial germination was determined every two hours for 12 h and indicated as germination percentage (%).

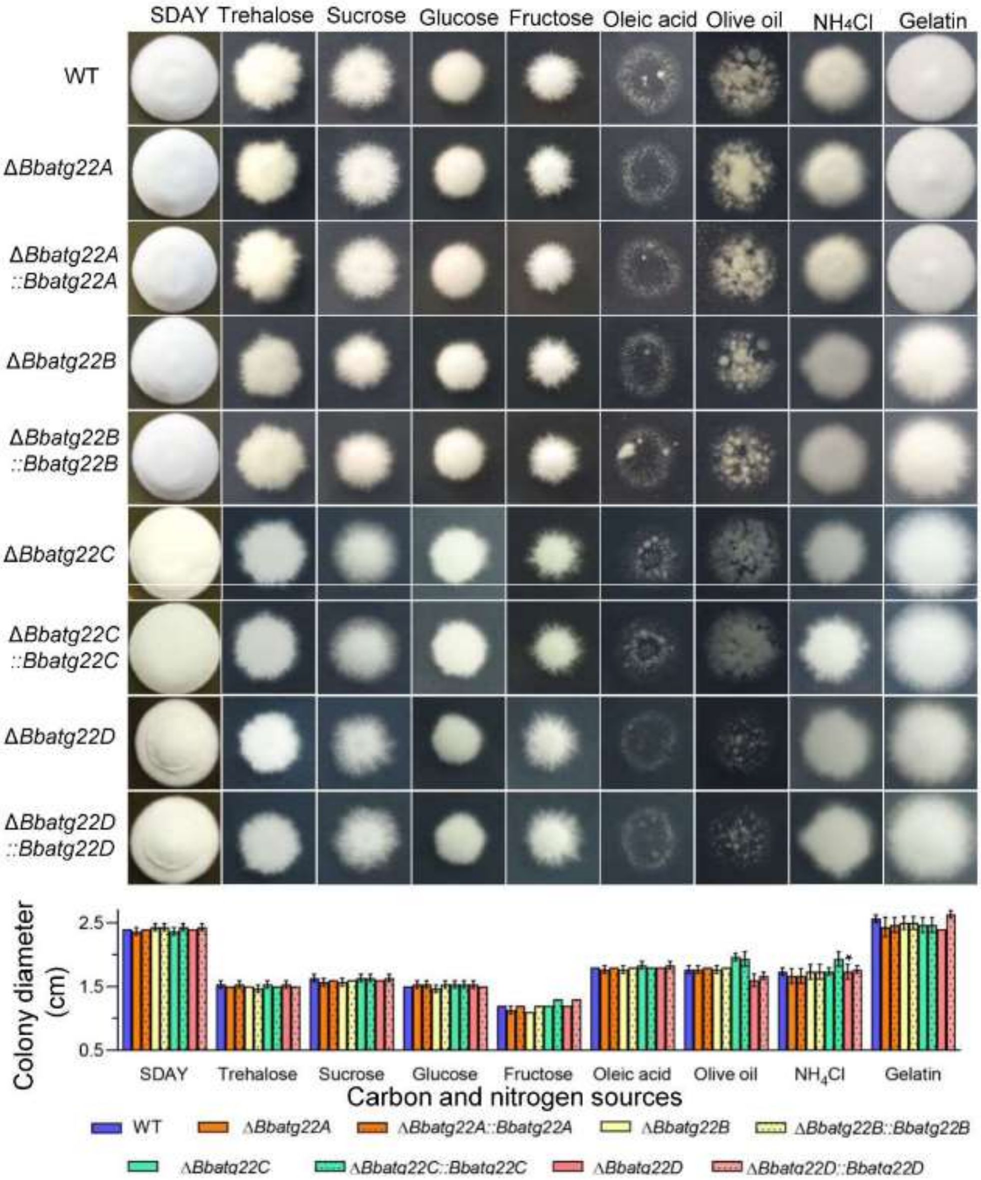

Figure S3. Effects of the gene loss on vegetative growth of *B. bassiana*.
On the basis of CZA, sucrose and NaNO3 were replaced with the indicated carbon and nitrogen sources, including treh alose, sucrose, glucose, fructose, oleic acid, olive oil, NH4Cl, and gelatin. SDAY plate was used as the control of nutrient-rich medium. The wild-type (WT), gene disruption and complementation mutant strains were cultured on SDAY plates till conidiation. Conidial suspension was inoculated on the plates. Colony diameter was measured after incubation of 7 d at 25 °C. There was no significant difference in colony diameter between the wild-type and gene disruption mutant strain.

Table S1. Primers used in this study.

**Primers**	**Paired sequences (5′–3′)***	**Purpose**
$P_{Atg22A}1$/$P_{Atg22A}2$	CATTCAATCACAAACACCTTCAAAATGCCGCCA AACGCGTCTCGCCCCA/CAGCTCCTCGCCCTTGC TCACCATACATCTTGCCGTCTCTTCCGAGTCG	Amplifying *BbATG22A* cDNA
$P_{Atg22A}3$/$P_{Atg22A}4$	CGAGCTGTACAAGTAACCCGGGGCCAGGGCAGT TGTCTTTCT/TTGGCTGCAGGTCGACGGATCCTTC GCGGCCAATGTCTTGTT	Amplifying 5′-fragment of *BbATG22A*

$P_{Atg22A}5/P_{Atg22A}6$	CGACCCATGGCTCGAGTCTAGACGACTTAGGCTCATTTATCCC/GGTGGTGGTGGCTAGCGTTAACTGTATCCGCCACAACTCCTT	Amplifying 3′-fragment of *BbATG22A*
$P_{Atg22A}7/P_{Atg22A}8$	ACAGTACACGAGGACTTCTAGAGCCAGGGCAGTTGTCTTTCT/GCCCTGCCCCTGAGAGGAATTCTGTTACAGCCCTGAGTCAAAGTA	Cloning the *BbATG22A* full ORF for gene complementation
$P_{Atg22A}9/P_{Atg22A}10$	TACAATACCGTCGGGCTGTTA/GACAAGTCCGAGGACATAGACG	Screening transformants
$P_{Atg22B}1/P_{Atg22B}2$	CATTCAATCACAAACACCTTCAAAATGATGGGGAGCAAGGCTTCTCGAGAT/CAGCTCCTCGCCCTTGCTCACCATTGCTGTGATAGTATTCTGAGTCTTCT	Amplifying *BbATG22B* cDNA
$P_{Atg22B}3/P_{Atg22B}4$	CGAGCTGTACAAGTAACCCGGGTTTAAAAGACTCGTCAAGAATA/TTGGCTGCAGGTCGACGGATCCGAAATGGGCTCAAGTTCGTATC	Amplifying 5′-fragment of *BbAtg22B*
$P_{Atg22B}5/P_{Atg22B}6$	CGACCCATGGCTCGAGTCTAGACCTGATTGCCTACCAGCTCACA/GGTGGTGGTGGCTAGCGTTAACCGCATCAACATCCGCACAAT	Amplifying 3′-fragment of *BbATG22B*
$P_{Atg22B}7/P_{Atg22B}8$	ACAGTACACGAGGACTTCTAGATTTAAAAGACTCGTCAAGAATA/GCCCTGCCCCTGAGAGGAATTCCGCATCAACATCCGCACAAT	Cloning the *BbAtg22B* full ORF for gene complementation
$P_{Atg22B}9/P_{Atg22B}10$	TTTCGTGAGCGATGCGTGAT/GACAAGTCCGAGGACATAGACG	Screening transformants
$P_{Atg22C}1/P_{Atg22C}2$	CATTCAATCACAAACACCTTCAAAATGGATTCTGTCCGGCACCCCGAGGA/CAGCTCCTCGCCCTTGCTCACCATTGCCGTCGTTGCAGCATCACTACTG	Amplifying *BbATG22C* cDNA
$P_{Atg22C}3/P_{Atg22C}4$	CGAGCTGTACAAGTAACCCGGGAAAACACTGGCGTAGTAACCG/TTGGCTGCAGGTCGACGGATCCGCTCAGCTTTCGCAAATACC	Amplifying 5′-fragment of *BbATG22C*
$P_{Atg22C}5/P_{Atg22C}6$	CGACCCATGGCTCGAGTCTAGATTGGTGGTAATGGTGGTGCT/GGTGGTGGTGGCTAGCGTTAACGTTTGAAAGTTCCAAGCCTGTA	Amplifying 3′-fragment of *BbATG22C*
$P_{Atg22C}7/P_{Atg22C}8$	ACAGTACACGAGGACTTCTAGACGAGAATGACCGTCTGGAAT/GCCCTGCCCCTGAGAGGAATTCGAGGGATGAATACAAGTAACCACA	Cloning the *BbATG22C* full ORF for gene complementation
$P_{Atg22C}9/P_{Atg22C}10$	GCGGCTTCTTTATGAACATT/GAAACCTGAGAGAGACTTGCTA	Screening transformants
$P_{Atg22D}1/P_{Atg22D}2$	CATTCAATCACAAACACCTTCAAAATGAGCTACCAAGGGCACGGAAATCGT/CAGCTCCTCGCCCTTGCTCACCATATTAGCCGATACCAGAGTCTTCTCCGCAT	Amplifying *BbATG22D* cDNA

$P_{Atg22D}3$/$P_{Atg22D}4$	CGAGCTGTACAAGTAACCCGGGACTGTATTATTAGCGGATGTCG/TTGGCTGCAGGTCGACGGATCCGTAATGATGTAGCGGTTTATGC	Amplifying 5′-fragment of *BbATG22D*
$P_{Atg22D}5$/$P_{Atg22D}6$	CGACCCATGGCTCGAGTCTAGAGCCTCGCCGACTACTCCAAT/GGTGGTGGTGGCTAGCGTTAACCCATCGTGATGCAGTTCCCT	Amplifying 3′-fragment of *BbATG22D*
$P_{Atg22D}7$/$P_{Atg22D}8$	ACAGTACACGAGGACTTCTAGAGAGGACTGTATTATTAGCGGATGT/GCCCTGCCCCTGAGAGGAATTCTCATTTCGTTTCACGGTGGG	Cloning the *BbAtg22D* full ORF for gene complementation
$P_{Atg22D}9$/$P_{Atg22D}10$	GAACTGTGGCTCATTTGAAGAA/AATGCGCCAAACAGGTAGAT	Screening transformants
$P_{Atg22A}11$/$P_{Atg22A}12$	TATCCACCACAATCTCCT/CTGTGACCAAGTCGTATT	qRT-PCR for *BbAtg22A*
$P_{Atg22B}11$/$P_{Atg22B}12$	GAAATTGGACCGACAAAT/AATCATAATCTGAGAATAACTGTA	qRT-PCR for *BbAtg22B*
$P_{Atg22C}11$/$P_{Atg22C}12$	TGCCTGCTCTATCTTCTC/GCGAATACTTGATGACCTC	qRT-PCR for *BbAtg22C*
$P_{Atg22D}11$/$P_{Atg22D}12$	TCCAAGGTGTCATTCAAA/ATCCACATCTGTCCATAC	qRT-PCR for *BbAtg22D*
18SF/18SR	TGGTTTCTAGGACCGCCGTAA/CCTTGGCAAATGCTTTCGC	Reference in qRT-PCR

*: The underlined region is required for homologous recombination during plasmid.
